# Supplementary material for: Predicting the Intention to Sort Waste at Home in Rural Communities in Lebanon: An Application of the Theory of Planned Behaviour
Source: Int J Environ Res Public Health. 2022 Jul 31;19(15):9383. doi: 10.3390/ijerph19159383 (PMC9368497; doi:10.3390/ijerph19159383)
Supplement: Supplementary file 1 [file ijerph-19-09383-s001.zip › ijerph-1827567-supplementary.pdf]

RES-Q questionnaire

(English and Arabic versions)

English	Arabic
**Satisfaction with waste management services**	**الرضا عن خدمات إدارة النفايات**
**How satisfied are you with the way waste is being collected in your community?**<br>(1 Extremely dissatisfied - 10 Extremely satisfied)<br>1---<br>--10	**ما مدى رضاك عن الطريقة التي يتم بها جمع النفايات في مجتمعك ؟**<br>(1 غير راضٍ أبداً - 10 راضٍ للغاية)<br>10---1
How satisfied are you with the way waste is being treated/disposed of in your community?<br>**(1 Extremely dissatisfied - 10 Extremely satisfied)**<br>1---<br>--10	**ما مدى رضاك عن طريقة التخلص من/ معالجة النفايات في مجتمعك ؟**<br>(1 غير راضٍ أبداً - 10 راضٍ للغاية)<br>10---1
**Recycling**	**إعادة التدوير**
**Do you separate organic waste from recyclable materials such as glass, plastic, cans & paper?**<br>• Yes<br>• No<br>• I prefer not to answer	**هل تفصل النفايات العضوية عن المواد القابلة لإعادة التدوير مثل الزجاج والبلاستيك والعلب والورق؟**<br>• نعم<br>• كلا<br>• أفضل عدم الإجابة
**What materials do you currently segregate?**<br>(Multiple options available)<br>• Plastic (carrier bag, bottle, yogurt pot)<br>• Aluminium (cans, foil, aerosol)<br>• Paper (e.g. books, newspapers, magazines, greeting cards)<br>• Glass (glass bottles, jars)<br>• Tires<br>• Electrical waste (batteries, mobile phones, computers, appliances, etc.)<br>• Textiles (clothes, shoes)<br>• Other metals (for example: iron)<br>• Other<br>• I prefer not to answer<br>What other materials do you currently segregate?<br>____________________	**ما هي المواد التي تفصلها حالياً؟**<br>(يمكنك إختيار أكثر من خيار)<br>• البلاستيك (كيس ، قنينة، وعاء اللبن)<br>• الألومنيوم (علب ، ورق ألومنيوم ، علب الهباء الجوي)<br>• ورق (مثل الكتب والصحف والمجلات وبطاقات المعايدة)<br>• الزجاج (الزجاجات ، الجرار)<br>• الإطارات<br>• النفايات الكهربائية (البطاريات والهواتف المحمولة وأجهزة الكمبيوتر والأجهزة وغيرها)<br>• المنسوجات (الملابس والأحذية)<br>• معادن أخرى (على سبيل المثال: الحديد)<br>• غير ذلك<br>• أفضل عدم الإجابة<br>ما هي المواد الأخرى التي تفصلها حاليا؟____________________

**Why don’t you recycle?**<br>(Multiple options available)<br>• I don’t know what I can recycle<br>• I don't know where to put the recyclables (I have no space)<br>• I have no time to separate the garbage<br>• I have no time to bring the recyclables to a drop-off point<br>• It requires too much effort<br>• Nobody is doing it in my village<br>• There is no system to collect the recyclables<br>• I do not care about recycling in this country<br>• If they paid me, I’d recycle<br>• Recycling doesn’t make a difference. So why doing it?<br>• I don't know<br>• Other<br>• I prefer not to answer<br>What are other reasons why you don't recycle?<br>________________	**لماذا□ تقوم بإعادة التدوير؟**<br>(يمكنك إختيار أكثر من خيار)<br>• □ أعرف ما هي المواد يمكنني إعادة تدويرها<br>• □ أعرف أين أضع المواد القابلة لإعادة التدوير (□ أملك مساحة كافية)<br>• □ أملك الوقت الكافي لفصل النفايات<br>• □ أملك الوقت الكافي لإيصال المواد القابلة لإعادة التدوير إلى المواقع المخصصة لجمعها<br>• يتطلب الكثير من الجهد<br>• □ أحد يفعل ذلك في بلدتي<br>• □ يوجد نظام لجمع المواد القابلة لإعادة التدوير<br>• لست مهتماً بموضوع إعادة التدوير في هذا البلد<br>• إذا دفعوا لي ، سأقوم بإعادة التدوير<br>• القيام بإعادة التدوير □ يحدث فرقا، فلماذا أفعل ذلك؟<br>• □ أدري<br>• غير ذلك<br>• أفضل عدم الإجابة<br><br>**ما هي الأسباب الأخرى التي تمنعك من إعادة تدوير النفايات؟**<br>________________________
**Why do you recycle?**<br>(Multiple options available)<br>• Recycling saves energy<br>• Recycling reduces landfills<br>• Preserves our resources and protects wildlife<br>• Recycling is good for the economy.<br>• Recycling helps our climate problems<br>• Other<br>• I don't know<br>• I prefer not to answer<br>**What are other reasons why you recycle?**<br>____________________	**لماذا تقوم بإعادة التدوير ؟**<br>(يمكنك إختيار أكثر من خيار)<br>• إعادة التدوير توفر الطاقة<br>• إعادة التدوير تقلل من المكبات العشوائية ومطامر النفايات<br>• تحافظ على مواردنا وتحمي الحياة البرية<br>• إعادة التدوير جيدة للاقتصاد<br>• إعادة التدوير تساعدفي حل مشاكلنا المناخية<br>• غير ذلك<br>• □ أدري<br>• أفضل عدم الإجابة<br><br>**ما هي الأسباب الأخرى التي تدفعك إلى القيام بإعادة تدوير النفايات ؟**________________
**<u>General attitudes about reducing, reusing, recycling</u>**	**<u>السلوكيات المتّبعة في ما يخص تخفيف استهلاك النفايات وإعادة استعمالها وإعادة تدويرها</u>**
There are different ways in which we can manage waste:<br>1. REDUCE- Avoid creating waste<br>2. REUSE – Use a product again or for another purpose<br>3. RECYCLE - Using materials again<br>4. RECOVER - Producing energy from waste	"هناك طرق مختلفة يمكننا من خلالها إدارة النفايات:<br>1- تخفيف استهلاك النفايات - تجنب إصدار النفايات<br>2-إعادة استخدام النفايات - استخدام المنتج مرة أخرى أو لغرض آخر<br>3-إعادة تدوير النفايات -استخدام المواد مرة أخرى<br>4 -استرجاع النفايات - إنتاج الطاقة من النفايات"

How important is for you to…<br>1 Not important at all - 10 Absolutely essential	**ما مدى أهمية التالي بالنسبة لك....**<br>1 ليس مهماً أبداً - 10 مهم جداً
**Reduce- Avoid creating waste**<br>**1---10**<br><br>**Reuse- Use a product again or for another purpose**<br>**1---10**<br><br>**Recycle- Using materials again**<br>**1---10**<br><br>**Recover- Producing energy from waste**<br>**1---10**	**تخفيف استهلاك النفايات - تجنب إصدار النفايات**<br>**10--1**<br><br>**إعادة استخدام النفايات - استخدام المنتج مرة أخرى أو لغرض آخر**<br>**10--1**<br><br>**إعادة تدوير النفايات-استخدام المواد مرة أخرى**<br>**10--1**<br><br>**استرجاع النفايات - إنتاج الطاقة من النفايات**<br>**10--1**
**<u>Attitudes</u>**	**<u>المواقف</u>**
**"Please rate the following behaviors according to the following couples of opposite adjectives:**<br><br>harmful vs. beneficial<br>good vs. bad<br>worthless vs. valuable<br>enjoyable vs. unenjoyable"	**يرجى تقييم السلوكيات التالية وفقًا للأزواج التالية من الصفات المعاكسة:**<br>ضارة مقابل مفيدة<br>جيد مقابل سيء<br>غير قيمة مقابل قيمة<br>ممتعة مقابل غير ممتعة
**Separate waste (e.g. organic from glass, plastic, cans, & paper) is…**<br>**1 Harmful - 10 Beneficial**<br>**1---10**<br><br>**1 Bad - 10 Good**<br>**1---10**<br><br>**1 Worthless - 10 Valuable**<br>**1---10**<br><br>**1 Unenjoyable - 10 Enjoyable**<br>**1---10**	**فصل النفايات (علي سبيل المثال العضوية من الزجاج والبلاستيك والعلب المعدنية والورق) هو**<br><br>**1 ضار -10 مفيد**<br>**10--1**<br><br>**1 سيئ- 10 جيد**<br>**10--1**<br><br>**1 غير قيم- 10 قيم**<br>**10--1**<br><br>**1 غير ممتع -10 ممتع**<br>**10--1**
**<u>Subjective Norms</u>**<br>**"How much do you agree or disagree with the following statements?**<br><br>**Think about the people who are important to you and that are similar to you."**	**<u>معايير شخصية</u>**<br>**ما مدى موافقتك أو عدم موافقتك على العبارات التالية؟**<br>**فكر في الأشخاص المقربين منك والمحيطين بك (عائلتك وأصدقائك).**<br>1 اعارض بشدة- 10 أوافق بشدة

1 Strongly Disagree; 10 Strongly Agree	
**Most people who are important to me approve that I**<br>**Separate waste (e.g. organic from glass, plastic, cans, & paper)**<br>**1---10**	**معظم الناس المقربين مني والمحيطين بي يوافقون على انني**<br><br>**أفصل النفايات (مثل العضوية من الزجاج والبلاستيك والعلب المعدنية والورق)**<br>**10---1**
**Most people like me are currently…**<br>**Separating waste (e.g. organic from glass, plastic, cans, & paper)**<br>**1---10**	**معظم الناس مثلي (عامة الناس) يقومون حاليًا ب**<br><br>**فصل النفايات (مثل العضوية من الزجاج والبلاستيك والعلب والورق)**<br>**10---1**
**Perceived Behavioral Control**<br>**How much do you agree or disagree with the following statements?**<br>1 Strongly Disagree; 10 Strongly Agree	**<u>إدراك التحكم السلوكى</u>**<br>**ما مدى موافقتك أو عدم موافقتك على العبارات التالية؟**<br>1 اعارض بشدة- 10 أوافق بشدة
**In the coming month, I am confident that I can**<br>**Separate waste (e.g. organic from glass, plastic, cans, & paper)**<br>**1---10**	**في الشهر المقبل، أنا على ثقة بأنني استطيع**<br><br>**فصل النفايات (مثل العضوية من الزجاج والبلاستيك والعلب المعدنية والورق)**<br>**10---1**
**In the coming month, it is totally up to me whether or not I can**<br>**Separate waste (e.g. organic from glass, plastic, cans, & paper)**<br>**1---10**	**في الشهر المقبل ، الأمر عائد لي تمامًا سواء استطعت أم لم استطيع**<br>**فصل النفايات (مثل العضوية من الزجاج والبلاستيك والعلب المعدنية والورق)**<br>**10---1**
**<u>Behavioral intention</u>**	**<u>النية السلوكية</u>**
**In the coming month, how likely are you going to…**<br>1 Very Unlikely - 10 Very likely<br>**Separate waste (e.g. organic from glass, plastic, cans, & paper)**<br>**1---10**	**في الشهر القادم ، ما مدى احتمال أن تقوم ب**<br>1 غير محتمل أبداً- 10 محتمل جداً<br><br>**فصل النفايات (مثل العضوية من الزجاج والبلاستيك والعلب المعدنية والورق)**<br>**10---1**
**How much do you agree or disagree with the following statements?**<br>1 Strongly Disagree; 10 Strongly Agree<br><br>**In the coming month, I am planning to…**<br>**Separate waste (e.g. organic from glass, plastic, cans, & paper)**	**ما مدى موافقتك أو عدم موافقتك على العبارات التالية؟**<br>1 اعارض بشدة- 10 أوافق بشدة<br>**في الشهر القادم ، أنا أخطط ل**<br><br>**فصل النفايات (مثل العضوية من الزجاج والبلاستيك والعلب المعدنية والورق)**<br>**10---1**

**1---10**	
**<u>Demographics</u>**	**<u>البيانات الديمغرافية</u>**
**Are you Female or Male?**<br>• Female<br>• Male	**هل أنت ذكر أم أنثى؟**<br>• أنثى<br>• ذكر
**What is the highest level of education you achieved?**<br>• Bachelor<br>• Master<br>• PhD<br>• Secondary<br>• Vocational<br>• Complementary<br>• Elementary<br>• No education<br>• I don't know	**ما هو أعلى مستوى تعليمي حصلت عليه؟**<br>• بكالوريوس<br>• ماجستير<br>• دكتوراه<br>• ثانوي<br>• مهني<br>• تكميلي<br>• ابتدائي<br>• □ شيء<br>• □ أدري
**Are you currently…**<br>• Single<br>• Engaged/in a relationship<br>• Married<br>• Divorced/Separated<br>• Widowed<br>• I prefer not to answer	**هل انت حاليا ...**<br>• غير مرتبط (ة)<br>• خاطب/ مخطوبة/ في علاقة<br>• متأهل(ة)<br>• منفصل (ة)/ مطلق (ة)<br>• أرمل (ة)<br>• أفضل عدم الإجابة
**Do you have children?**<br>• Yes<br>• No<br>• I prefer not to answer	**هل لديك أطفال؟**<br>• نعم<br>• كلا<br>• أفضل عدم الإجابة
**How many children younger than 18 years old do you have?**<br>________________________	**كم عدد الأطفال الذين تقل أعمارهم عن 18 عامًا؟-________________**
**Including yourself, how many people are living in your household, excluding any newborn infants?**<br>________________________	**كم عدد الأشخاص الذين يعيشون في منزلك بما فيهم أنت، باستثناء الأطفال حديثي الو□دة؟**<br>________________
**How many rooms does your household have, excluding kitchen and bathrooms?**<br>________________________	**كم عدد الغرف في منزلك ، باستثناء المطبخ والحمامات؟**<br>________________________
